# The use of reimbursement data for timely monitoring of vaccination coverage: the example of human papillomavirus vaccine following public concerns about vaccine safety

**DOI:** 10.1186/s12889-015-2575-7

**Published:** 2015-12-12

**Authors:** Laure Fonteneau, Marine Ragot, Isabelle Parent du Châtelet, Jean-Paul Guthmann, Daniel Lévy-Bruhl

**Affiliations:** Institut de Veille Sanitaire (InVS), Saint-Maurice, France; European Programme for Intervention Epidemiology Training (EPIET), European Centre for Disease Prevention and Control, (ECDC), Stockholm, Sweden

**Keywords:** Vaccination, Papillomavirus, France, Vaccine coverage, Rreimbursement data

## Abstract

**Background:**

Since 2011 public concerns about Human Papillomavirus (HPV) vaccination safety and efficacy arose in France. We explored the relevance of using vaccines reimbursement data to assess the impact of those public concerns on vaccination coverage.

**Methods:**

We used the Permanent Sample of Beneficiaries which was, at the time of the study, a representative sample of 1/97^th^ health insurance beneficiaries of the main Social Security scheme, the General Health Insurance Scheme, covering approximately 77 % of the French resident population. We estimated HPV vaccination coverage among girls born between 1995 and 1999 at their 15^th^, 16^th^ and 17^th^ birthday.

**Results:**

The coverage for complete vaccination among 16 years old girls decreased from 26.5 % in the first semester of 2011 to 18.6 % in the first semester of 2014.

**Conclusions:**

HPV vaccination coverage was already low in 2011 and continued to decrease thereafter. Vaccines reimbursement data allowed us to reactively monitor the impact of the controversy on vaccination coverage and design counteracting measures.

## Background

In 2012, cervical cancer was the 11th most common cancer in women in France with 3028 estimated new cases and the 12^th^ in terms of mortality with an estimated 1 102 deaths [1]. The human papillomavirus (HPV) is a necessary cause of cervical cancer [2]. In addition to the opportunistic screening strategy based on Pap smears recommended every 3 years for women 25 to 65 years old, HPV vaccination was introduced in the immunisation schedule in 2007. Up to 2013, vaccination was recommended for girls aged 14 years old, with a catch-up offered for girls aged 15–23 years old who had not started their sexual life or in the year following their sexual life debut [3]. Three doses were required for a complete vaccination course [4].

In summer 2011, articles questioning HPV vaccine safety were published in the lay media, telling the stories of teenagers having developed neurological manifestations after HPV vaccination. A group of physicians opposed to HPV vaccination organized several manifestations including a public meeting with members of parliament, questioning the relevance of vaccination in addition to pre-cancerous lesions screening. In October 2011, the High Council for Public Health issued a statement reaffirming the favorable risk/benefit balance of HPV vaccination [5]. In autumn 2013 the debate was reopened with the decision of a Regional commission for compensation for medical accidents which concluded to the partial responsibility of the HPV vaccine in the development of multiple sclerosis in a vaccinated adolescent girl. This event was reported by the media.

Up to recently, the only routine source of vaccination coverage among teenagers were the national school surveys performed every six years at 15 years of age [6, 7]. Although providing reliable results, they were limited by their poor timeliness. We therefore explored the relevance of using vaccine reimbursement data to closely monitor the impact of public concerns on HPV vaccination coverage.

## Methods

HPV vaccination is almost exclusively administered in France through the private sector. Patients purchase the vaccine at pharmacies on a medical prescription. Virtually the whole French population is covered by the National Health Insurance Scheme (NHIS) which covers 65 % of the price of the vaccine. A complementary insurance covers the remaining 35 % of the vaccine cost for over 90 % of the population. This reimbursement data are entered into a single database, the National Health Insurance Information System, which contains anonymous and comprehensive data on the health care consumption of the entire French population.

In order to give access to a manageable database to Public Health institutions, a sample of this huge database, called the Permanent Sample of Beneficiaries (PSB), was established in 2005. Through a decree signed by the Minister of Health in 2005, the National Institute for Public Health Surveillance (InVS) was given access to the PSB, together with a restricted list of public institutions. At that time, the PSB was a representative sample of 1/97^th^ health insurance beneficiaries covered by the main scheme, the General Health Insurance Scheme, covering approximately 77 % of the French resident population. It included about 500,000 persons, followed for all their health care consumptions for a period of at least 20 years [8, 9].

We use the PSB to bi-annually monitor HPV vaccination coverage. The current analysis presents vaccination data for girls born between 1995 and 1999 and aged 15 years between January 2010 and June 2014. We estimated coverage at their 15^th^, 16^th^ and 17^th^ birthday. We extracted data related to HPV vaccines purchased between July 2007 (date of first admission to reimbursement by the NHIS of a HPV vaccine) and June 2014. We extracted data from the date of the first admission to reimbursement as some girls may have been vaccinated before 14 years of age, the marketing authorization of both vaccines having been granted for girls from 9 years of age. To take into account a possible delay between vaccine purchase and availability of the information in the PSB, we extracted the data in October 2014. For each selected individual, we extracted his/her vaccine history (number of doses reimbursed and age at delivery). The age-specific vaccination coverage was calculated by dividing, for each dose of vaccine, the number of doses reimbursed by that age, by the number of eligible girls of that age in the PSB. In order to assess more precisely the potential impact of media on the HPV vaccination coverage we retrospectively estimate the vaccination coverage by quarter.

A decree dated 20th June 2005 gives to accredited InVS staff access to anonymized individual PSB data. This decree was issued after receiving a positive opinion from the French Data Protection Agency (CNIL). The author, who did the extraction and analysis, is accredited for PSB database access.

## Results

**Fig. 1:**
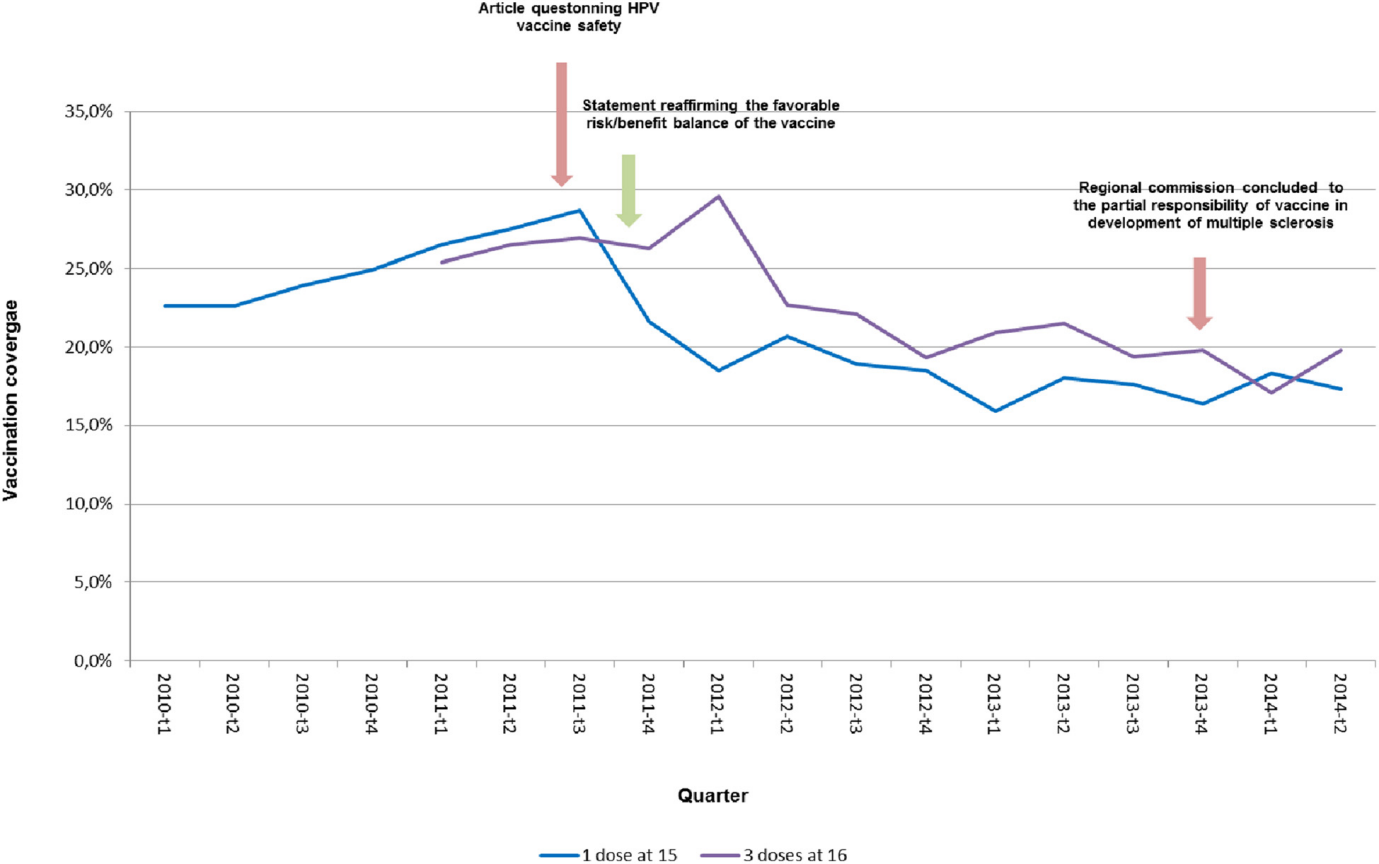
Vaccination coverage with one dose of HPV vaccine at 15^th^ birthday and three doses at 16^th^ birthday, by quarter periods, January 2010–June 2014

The vaccine coverage for at least one dose at the 15^th^ birthday varied very little from 23.7 % (95 % confidence interval, CI [20.9–26.5]) to 26.5 % (95 % CI [24.1–28.9]) between 2010 and 2011. It decreased from 25.6 % (95 % CI [23.3–27.9]) in the second semester of 2011 to 16.3 % (95 % CI [14.3–18.3]) in the first semester of 2013 and has remained below 18 % since (Table 1 and Fig. 1).

**Table 1:** HPV vaccination coverage (%) and its 95 % confidence interval at 15^th^, 16^th^ and 17^th^ birthday as of June 30 2014, France

Half year of birthday	2010	2010	2011	2011	2012	2012	2013	2013	2014
	s1	s2	s1	s2	s1	s2	s1	s2	s1
n	877	914	1316	1340	1431	1430	1323	1408	1404
1 dose at 15	23.7 [20.9–26.5]	24.7 [21.9–27.5]	26.5 [24.1–28.9]	25.6 [23.3–27.9]	19.8 [17.1–21.9]	17.5 [15.5–19.5]	16.3 [14.3–18.3]	17.5 [15.5–19.5]	17.7 [15.7–19.7]
3 doses at 16	–	–	26.5 [23.6–29.4]	27.4 [24.5–30.3]	28.2 [25.8–30.6]	24.2 [21.9–26.5]	22.0 [19.9–24.1]	20.1 [18.0–22.2]	18.6 [16.5–20.7]
3 doses at 17	–	–	–	–	31.2 [28.8–33.6]	30.3 [27.9–32.7]	32.0 [29.5–34.5]	27.2 [24.9–29.5]	26.3 [24.0–28.6]

**Fig. 2:**
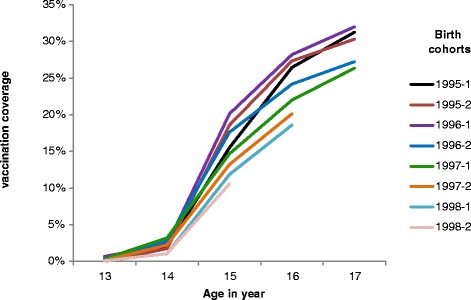
Cumulative vaccination coverage for 3 doses of HPV vaccine according to age and half year of birth. France

The vaccine coverage for the full series on the 16^th^ birthday varied very little from 26.5 % (95 % CI [23.6–29.4]) in the first half of 2011 to 28.2 % (95 % CI [25.8–30.6]) in the first half of 2012. It decreased from 28.2 % (95 % CI [25.8–30.6]) in the first semester of 2012 to 18.6 % (95 % CI [16.5–20.7]) in the first semester of 2014. Coverage slightly increased between the 16^th^ and 17^th^ birthday through a limited catch up. However the coverage among girls aged 17 years old decreased from 31.2 (95 % CI [28.6–33.6]) to 26.3 % (95 % CI [24.3–28.6]) from the first semester of 2013 to the first semester of 2014 (Table 1). For girls born after 1996, the 3 doses vaccination coverage at any age decreased for successive birth cohorts (Fig. 2).

## Discussion

Even for the 1995 birth cohort who reached 16 years of age at the time of the first publications in the lay media, the coverage at 16 years was below 30 %. The one dose HPV vaccine coverage dropped after the first publication from 25.6 % at the end of 2011 to 19.8 % at the beginning of 2012. We did not observe a second drop in vaccination coverage after the 2013 decision of a Regional commission for compensation for medical accidents in favor of the partial responsibility of the HPV vaccine in the development of multiple sclerosis in an adolescent girl. At this time the vaccination coverage was already very low (17.5 % for 1 dose) which could explain that it did not decreased further. Unfortunately, in 2011, the positive statement from the High Council for Public Health was not reported in the media and therefore could not impact favorably the HPV vaccination coverage. The social concerns related to the vaccine safety profile and the relevance of HPV vaccination in addition to screening are likely to have negatively affected HPV vaccination coverage and contributed to the very low level observed in 2014.

We were able to closely monitor the vaccination coverage trends through the use of vaccine reimbursement data. This new method represents an innovative approach to provide timely updated reliable vaccination coverage estimates. The time between actual delivery of the vaccine by the pharmacist and availability of the information in the PSB is only around 1 month. Beneficiaries are selected totally randomly. Indeed the beneficiaries with a strictly secret value of their National Identity Register (NIR) control key number (going from 1 to 97) are selected and included in the PSB whether they have consumed care or not. The validity of the PSB as a vaccine coverage measurement tool was previously validated through the comparison of the mumps measles and rubella (MMR) vaccination coverage estimated through the PSB and through the Child Health Certificate (CHC) which is our reference tool. MMR vaccine coverage estimates for one dose at 24 months for the 2010 birth cohort was very close: 91.4 % from PSB and 90.5 % through the CHC [10]. Our PSB estimates agree well with the few available other data. Our 23.7 % coverage for one dose among girls aged 15 years old in 2010 is consistent with the results of a school survey in which 24.7 % of girls aged on average 15 years old in 2009 had received one dose of HPV vaccine [5]. Our estimated coverage for complete vaccination of 24.2 % among girls aged 16 in 2012 is also consistent with the results of a survey in which 23.6 % of girls aged 15 years old in 2012 were fully immunized against HPV [11]. To our knowledge, except some experiences in Germany [12, 13], reimbursement data has not been used for the estimation of vaccination coverage in other European Countries [14]. It represents a cheaper and easier alternative to ad hoc surveys which can be very resource consuming. This source of data is particularly useful to monitor coverage in age groups such as teenagers usually not targeted by vaccination strategies and for which routine vaccination monitoring tools are lacking in many countries.

The use of PSB has some limitations. First, our analysis focused on the beneficiaries of the General Health Insurance Scheme covering only 77 % of the population and no data comparing the vaccination attitudes of the population according to their insurance coverage were available until recently. However, since 2011, data from two other schemes, covering an extra 10 % of the population, have been progressively integrated in the PSB. Vaccination coverage for one dose among 15 years old girls born in 1998 covered by these schemes, was 18.9 % (95 % CI [12.9–24.9]) and 18.5 % (95 % CI [12.6–24.3]), respectively, consistent with our estimation (95 % CI (17.8 % [16.4–19.2]). We were unable to estimate vaccination coverage after the age of 17 years because at this age, many girls become students and are no longer registered in the General Health Insurance Scheme. Limited information on factors that could have influenced the vaccine coverage is available in the PSB. However, the proportion of individuals registered in the PSB and benefiting from the Universal Health Care Coverage, which gives full free access to care to the most disadvantaged ones, did virtually not change during the study period. This indicator can be considered as a proxy of the socio-economic status of the PSB beneficiaries. Furthermore, the vaccination offer did not change during the study period.

Last, purchased vaccines may not all be administered and some persons may purchase their vaccine without claiming for reimbursement. There is no data to estimate the frequency of such situations, but we believe this to be very rare, especially for expensive vaccines such as HPV ones.

Some measures have been taken to try to improve the HPV vaccination coverage. A new report summarizing internationally available evidence regarding the safety, effectiveness and impact of HPV vaccination was prepared by the Public Health Council (HCSP) and published on its website on September 2014 to address the controversy [15]. To the same end, the French Institute for Health Promotion and Education has developed a community management plan for HPV vaccination to be implemented in 2016 in order to give official answers to concerns raised and questions asked on internet forums. Changes in the immunization schedule aiming at increasing the compliance to HPV vaccination recommendations has been made in 2014: the target age for routine vaccination has been enlarged from 14 to 11–14 years and the number of doses needed in this age group has been reduced from three to two [16].

## Conclusions

In conclusion HPV vaccination coverage was very low in France in 2011 and decrease afterwards probably in relation with media coverage regarding potential safety issues of the vaccine. The Permanent Sample of Beneficiaries was particularly useful to rapidly assess the impact of public concerns on HPV vaccination coverage. A new vaccine coverage assessment will be made by end-2015 and will allow measuring the impact on the recently implemented countermeasures on HPV vaccine coverage. 

## Abbreviations

HCSP: Public Health Council
HPV: Human papillomavirus
InVS: National Institute for Public Health Surveillance
NHIS: National Health Insurance Scheme
PSB: Permanent Sample of Beneficiaries (PSB)
HCSP: Public Health Council
